# Surgical robotic systems: What we have now? A urological perspective

**DOI:** 10.1002/bco2.31

**Published:** 2020-08-19

**Authors:** Ahmad Almujalhem, Koon Ho Rha

**Affiliations:** ^1^ Department of Urology Mubarak Alkabeer Hospital Safat Kuwait; ^2^ Department of Urology Yonsei University Severance Hospital Seoul South Korea

## Abstract

**Introduction:**

The coming decade will see the emergence of many surgical robotic systems that need to prove their cost‐effectiveness and clinical usability to gain the trust of robotic surgeons worldwide. Herein, we provide a concise review of the currently available robotic systems. Since the da Vinci Surgical System's patent expired and its market monopoly ended, many robotic surgical systems have, and will continue to, enter the market. Central to this is the challenge of gaining the trust of robotic surgeons in a cost‐effective manner. However, the cumulative robotic surgical experience of Intuitive Surgical over these years—which has proven itself clinically and technically—is a great challenge for new surgical robots.

**Methods:**

This was a non‐systematic review of the literature, conducted through the PubMed search engine, using the following words: “Da Vinci,” “robotic surgical system,” and “new robotic surgical device.” Further information was obtained from the robotic system companies’ official websites and press releases.

**Conclusions:**

The open robotic market carries great challenges for new robotic surgical systems, especially when following well‐established da Vinci Surgical System. Surgeons’ trust, clinical publications, technical support, and market distribution all represent separate challenges that require address.

## 1 INTRODUCTION

The da Vinci Surgical System was introduced in 1999 (Intuitive Surgical Inc., Mountain View, USA), and was approved by the US Food and Drug Administration (FDA) in 2000.^1^ The da Vinci robotic‐assisted laparoscopic surgery—with the help of enhanced 3D vision, new ranges of movement of laparoscopic tools, and highly responsive robots—ushered in a new era in minimally invasive surgery. So far, four generations of da Vinci have been introduced to the surgical field over the past 20 years.^2^, ^3^, ^4^, ^5^ The da Vinci's established intuitive surgical, clinical, technical, and marketing experience is the greatest challenge for all new surgical robot companies. Many hospitals will not be able to accommodate any more robots in their operation theaters, which is a marketing challenge for the new robotics companies. The clinical applications of patented new surgical robotic systems are limited, which needs to be overcome in the coming years.^6^, ^7^, ^8^ The annual revenue of the robotic surgery industry has reached around three billion dollars, with an expected annual growth of 15% in the coming years.^9^ Sharing in this growth is strong motivation for new robotic companies. With this new robotic era, we expect to see more robust competition among these robotic companies in the development and marketing of their new robots.^10^ The aim of this paper was to comprehensively review the emerging new robotic systems.

## 2 METHODS

A thorough non‐systematic literature review was performed using the PubMed electronic search engine. The following words were searched: “Da Vinci robot system” (n = 990), “robotic surgical system” (n = 6,303), and “new robotic surgical device” (n = 950). Study selection criteria were review articles, preclinical studies, and first clinical trials of these robotic surgical systems. Additional information was obtained from each robotic system company's official website and from press release articles. The included figures were sourced from pictures previously published in medical journals. This review focuses on robotic systems that have urological or other potential applications.

## 3 ROBOTIC SURGICAL SYSTEMS

The robotic systems are summarized in Table 1.

**TABLE 1:** Robotic surgical systems summary

Robot	Uses	Port	Robot Cart	Arm	Console	Controller	Enhanced imaging	Vision	Haptic feedback	Eye tracking	Instrument	Arm DOF	Instrument DOF	Cost (Million $)
DV Xi	MIS	MP	Single	4	Close	Joystick	FireFly	3D HD	No	No	10 uses	3	7	1–1.5
DV SP	MIS	SP	Single	1	Close	Joystick	FireFly	3D HD	No	No	10 uses	Rotational	Flexible	2
	NOTES													
	LESS													
Senhance	MIS	MP	Multiple	4	Open	Joystick	NOVADAQ	3D HD	Yes	Yes	Unlimited	3	7	1.3
							PINPOINT							
Revo‐i	MIS	MP	Single	4	Close	Joystick	No	3D HD	Yes	No	20 uses	3	7	NA
Micro Hand S	MIS	MP	Single	4	Close	Joystick	No	3D HD	No	No	NA	3	7	NA
SPORT	MIS	SP	Single	1	Open	Hand‐controller	No	3D HD	NA	NA	NA	Rotational	Flexible	NA
	NOTES													
	LESS													
Versius	MIS	MP	Multiple	4	Open	Joystick	No	3D HD	NA	NA	NA	3	7	Managed‐service contract system
MiroSurge	MIS	MP	Multiple	3	Open	Joystick	No	3D HD	Yes	NA	NA	3	7	NA
			Table‐mounted											
Bitrack	MIS	MP	Single	3	Open	Joystick	No	3D HD	NA	NA	NA	3	7	NA
Medicaroid	MIS	MP	Single	4	Open	Joystick	No	3D HD	NA	NA	NA	3	7	NA
Verb	MIS	MP	Single	4	Open	Joystick	NA	3D HD	NA	NA	NA	NA	NA	NA
SurgiBot	LESS	SP	Single	1	No console	Laparoscopic	NA	3D HD	NAP	NAP	NA	Rotational	Flexible	NA
						Handle								
PROCEPT	NOTES	NAP	Single	1	No console	NAP	Real‐time US	HD	NAP	NAP	NA	NAP	NAP	NA
EMARO	MIS	SP	Single	1	No console	Pneumatic	NAP	HD	NAP	NAP	NA	Rotational	NAP	NA
						Gyroscope								
Roboflex	NOTES	NAP	Single	1	Open	Joystick	NAP	HD	NA	NAP	NAP	Rotational	Flexible	NA

Note

DOF, degree of freedom; DV, da Vinci; LESS, laparoendoscopic single port surgery; MP, multi‐port; NA, no available data; NAP, not applicable; NOTES, natural orifice transluminal endoscopic surgery; SP, single port.

## 4 DA VINCI SURGICAL SYSTEM

**FIGURE 1:**
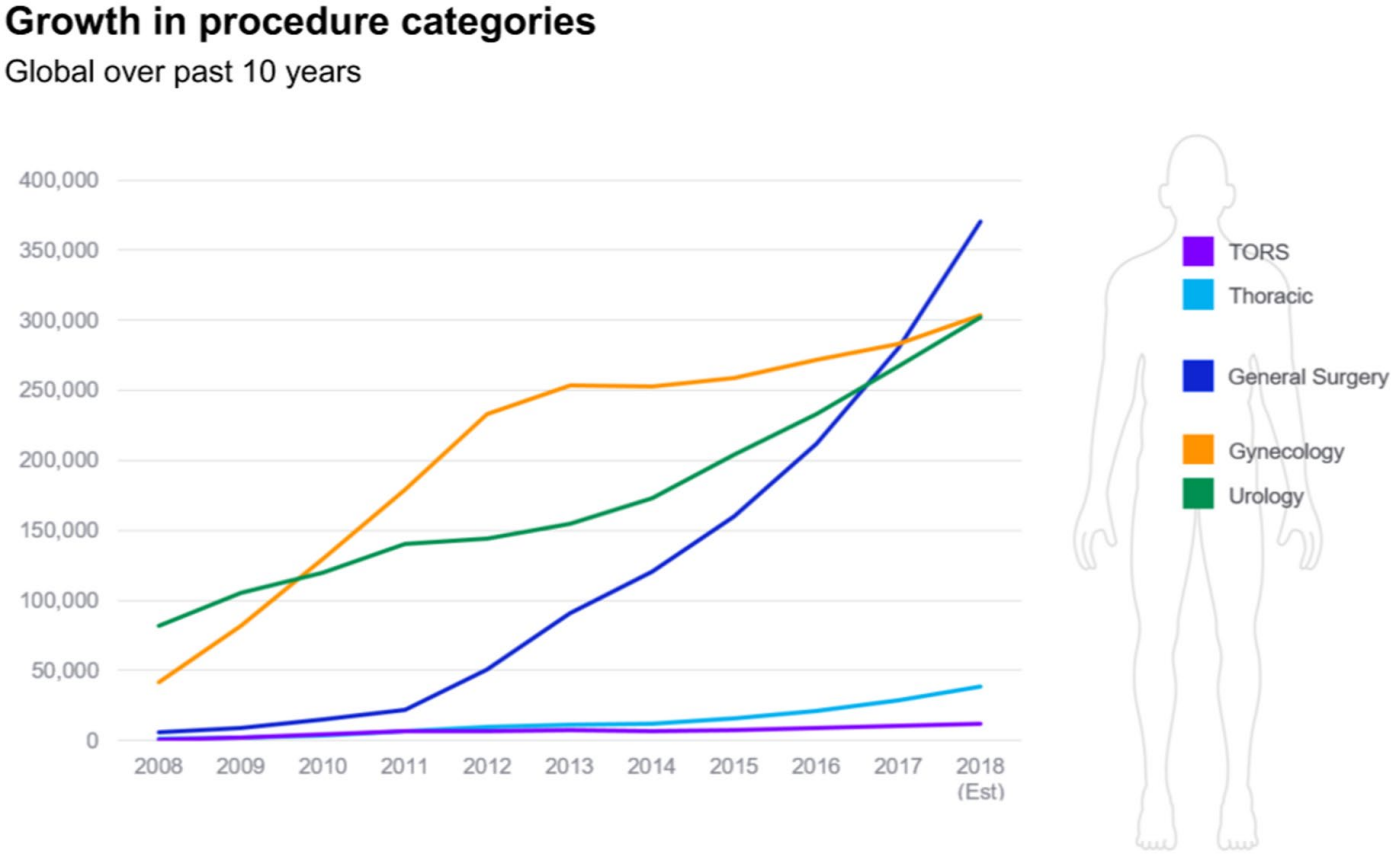
Robotic growth over surgical specialties^2^

The introduction of the da Vinci surgical system revolutionized many surgeries over many surgical specialties. The da Vinci system also created a new surgical history milestone: from open surgery, to laparoscopic and endoscopic surgery, to robot‐assisted surgery. The adoption of robotic surgery has grown rapidly over all surgical specialties (Figure 1).^11^

The long surgical innovation patency period gave intuitive surgery and elevated stature in the robotic surgical system market. Many surgical platforms were developed, as was trust from surgeons, patients, and health authorities. Additionally, there have been extensive clinical publications on top of a strong marketing strategy with well‐distributed distributors across the globe. Over the last 20 years, approximately five million surgeries have been performed with da Vinci Robots,^12^ which has given them a massive cumulative experience and competitive advantage.

**FIGURE 2:**
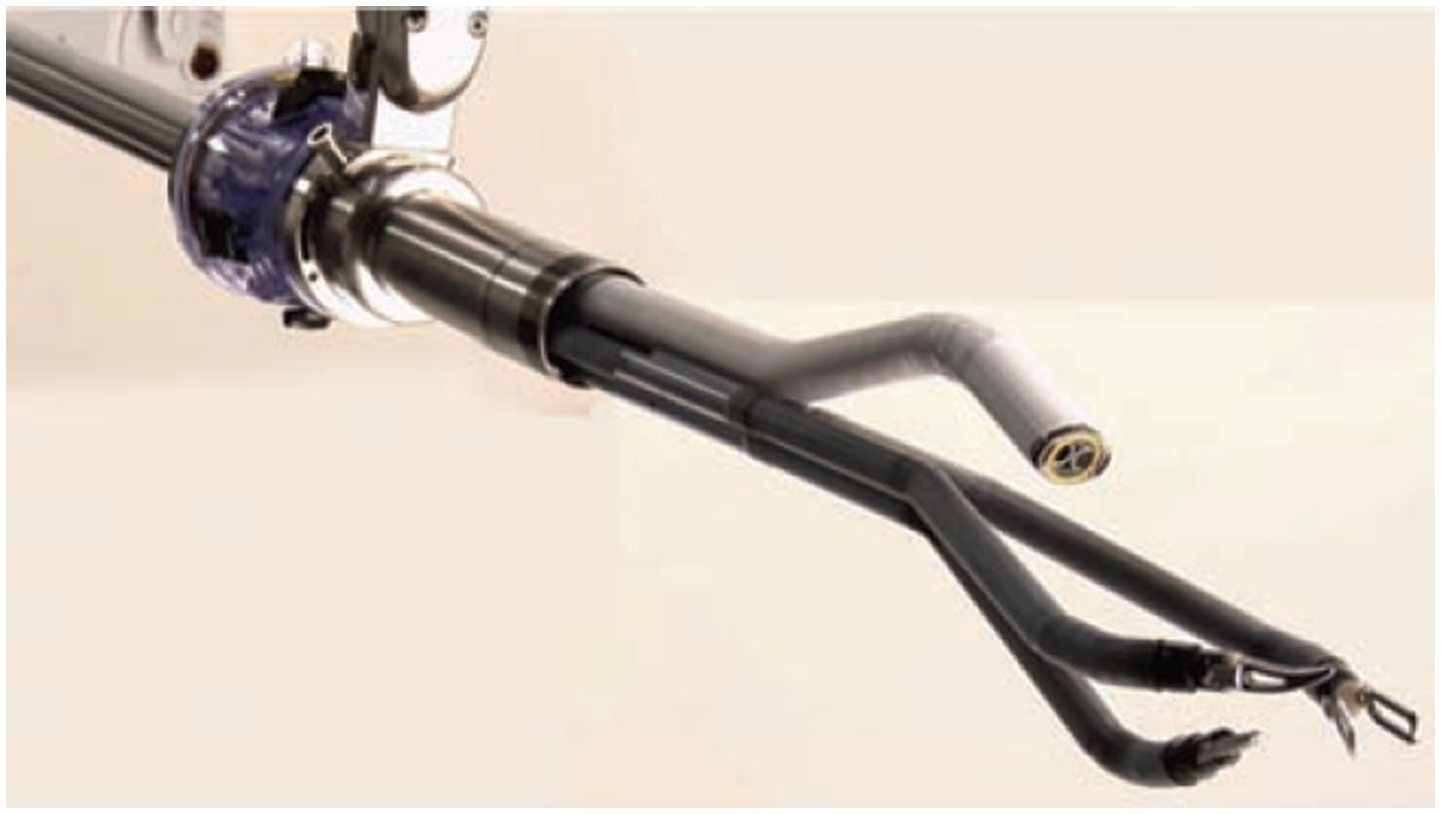
SP da Vinci robot^3^

The Da Vinci surgical system solved several challenges of standard laparoscopic surgery including improved 3D/HD vision, better dexterity, seven degrees of motion (DOF) by the EndoWrist system, and effective simulation training software. Intuitive surgery brought to surgeons da Vinci S, Si, Xi, X, and the gamechanger of their SP (single port) da Vinci Robotic system (Figure 2).^13^

## 5 NEW ROBOTIC SURGICAL SYSTEMS AND COMPANIES

### 5.1 Senhance (Telelap ALF‐X)

The recently (approved October 2017) FDA‐approved ALF‐X (Senhance; Trans‐Enterix®, Morrisville, USA) is a new multiport robotic system. Fanfani et al reported 80 hysterectomy cases from October 2013 to May 2014 with the ALF‐X system.^14^ For urology, a porcine model of robot‐assisted partial nephrectomy has been reported.^15^ It has the advantage of totally independent surgical arms, haptic feedback, and eye tracking systems.^16^, ^17^ Because of independent surgical arms, this system requires a spacious operative room.

### 5.2 Revo‐i®

**FIGURE 3:**
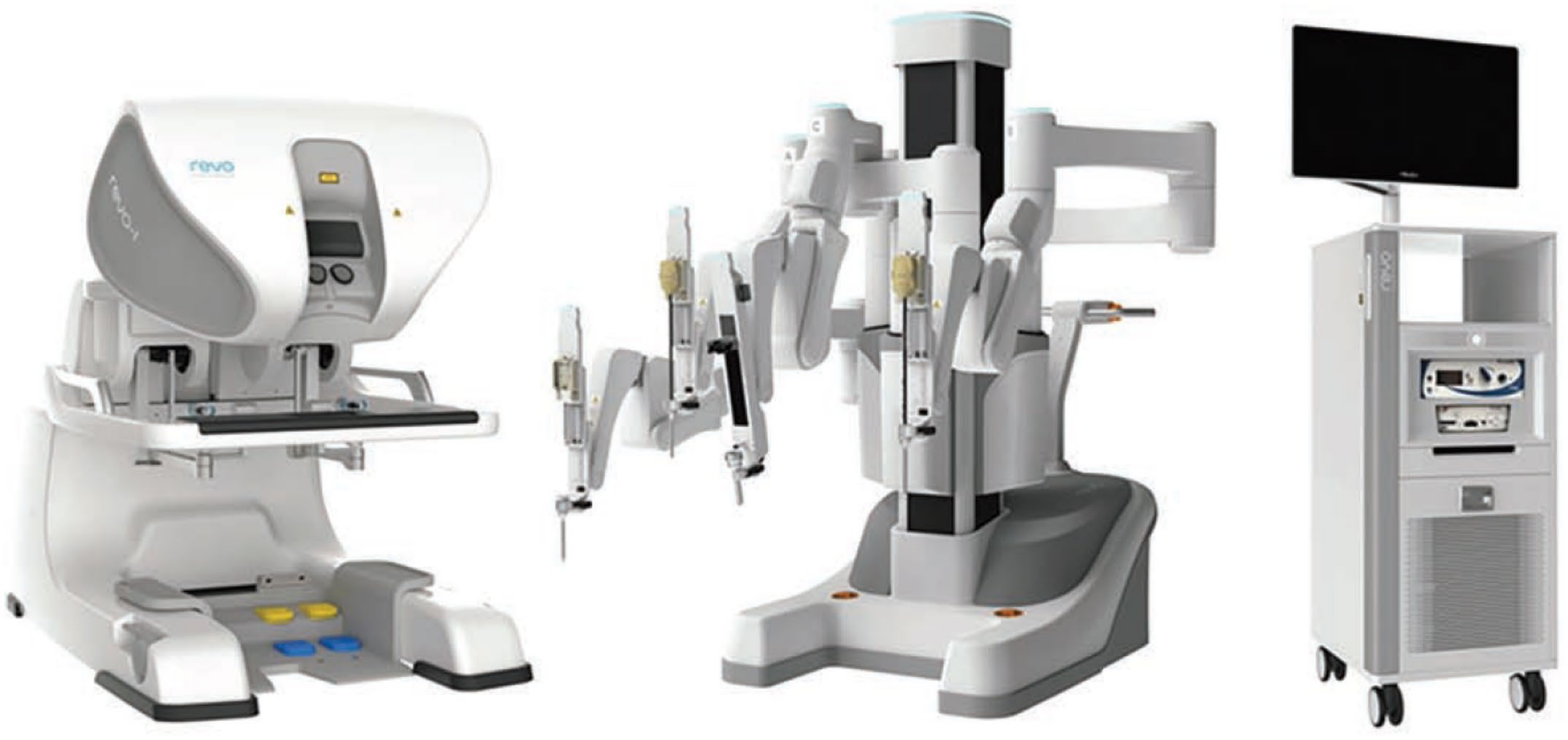
Revo‐I robot^3^, ^4^, ^5^

Revo‐i® (Mere company Inc., Yongin, South Korea) is the first Korean robotic system, and received Korean FDA approval for clinical use in August 2017. Revo‐i® has a control console, a four‐arm robotic cart, a vision cart with HD quality, and multi‐use endoscopic instruments (Figure 3).^13^, ^18^, ^19^ It has a design similar to that of the da Vinci Si robot. Abdel Raheem et al reported their porcine model preclinical study in 2016,^8^ and a clinical trial of robotic cholecystectomy in 2017.^20^

### 5.3 Micro Hand S

**FIGURE 4:**
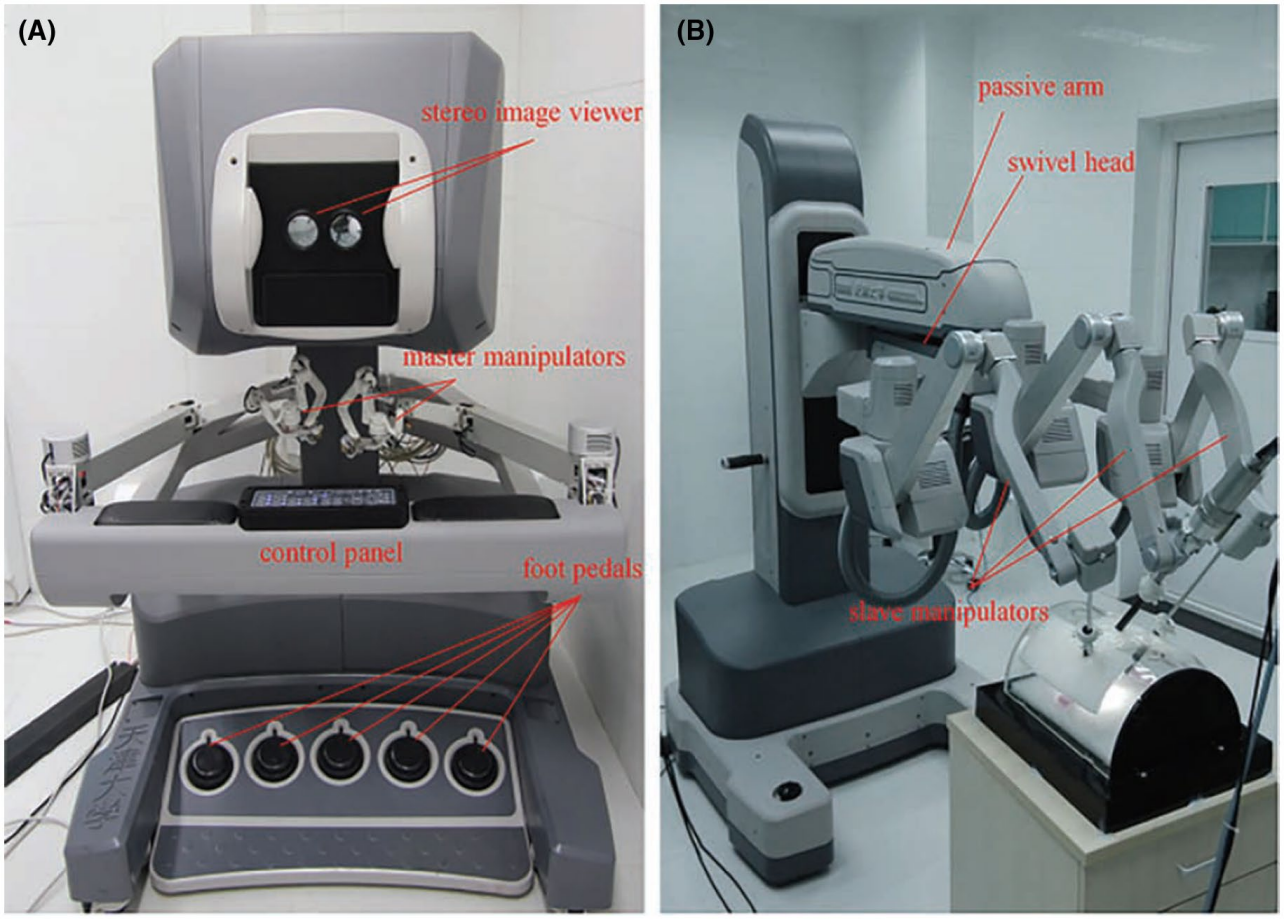
Micro Hand S robot^6^

The Micro Hand S first Chinese robotic system was developed by Tianjin University in collaboration with Central South University in 2013.^21^ It is similar to the da Vinci Si robot (Figure 4).^22^ In 2014, the first clinical trial was reported by Yi et al, who treated patients with gastric perforations and two patients with acute appendicitis.^22^

### 5.4 SPORT

The Single Port Orifice Robotic Technology (SPORT) was developed by the Titan Medical Company (Toronto, Canada).^23^ It has an open console system that supports HD/3D vision with a multi‐articulated robotic instrument. SPORT has been used to perform robotic single‐port partial nephrectomy using an animal model,^10^ but has not been used in human clinical studies. The company will have a challenging market, as the SP da Vinci Robot has already launched in multiple countries and was featured in a handful of clinical publications.

### 5.5 Versius

Versius (Cambridge Medical Robotics, Cambridge, UK) was created following the idea of independent robotic arms with separate functional units.^24^ It has an open console design. The robotic arm consists of three joints which act as the shoulder, elbow, and wrist. These joints provide a more human‐like range of arm movements. The creators of Versius also introduced a haptic feedback system. The company adopted a different marketing strategy in the form of a managed‐service contract system.^25^ without an upfront capital payment. This should attract mid‐range to small hospitals to add robotic surgeries to their existing services.

### 5.6 MiroSurge

MiroSurge (Medtronic, Minneapolis, USA) is an operative table mounted robot, as the robotic arms are attached to the operation table. The developers used the concept of an open console with an autofocus feature. MiroSurge is a micromotor driven instrument with a wide range of motion that also provides tactile feedback. We are still waiting for their clinical trials and applications to further assess this system's potential. The company was planning to lunch clinical studies in India, but does not appear to have done so yet.^26^, ^27^, ^28^

### 5.7 Bitrack

A prototype of the Bitrack System (Rob Surgical Systems S.L., Barcelona, Spain)^29^ was introduced in 2015. This was followed by a clinical validation phase to obtain European market approval. As with all new robotic systems, no sufficient clinical data exist to affirm the feasibility or superiority of this system to currently available robots. Other details of the system have been secured from public access.

### 5.8 Medicaroid

Medicaroid (Kobe, Japan) was introduced in 2016. It was developed by a company in Silicon Valley consisting of a collaborative group of Sysmex and Kawasaki.^30^, ^31^ Medicaroid is another table‐mounted robot that consists of three arms and an operating console.^10^ We are still waiting for their clinical trials to begin.

### 5.9 Verb

Verb Surgical, established in 2015, is the result of the collaboration between Google and Johnson & Johnson.^32^ They have introduced artificial intelligence (AI) to robotic systems with additional feedback systems to operating surgeons. The company's long‐term plan is to move toward a new era of robotic‐guided (rather than robot‐assisted) surgery. Over the long‐term, the company seeks to develop robotic surgery technology, fully performed by robots. To this end, no detailed information is available publicly about either the robot system or design. However, such information is anticipated in the near future, considering the collaboration of high‐tech and well‐established innovative surgical companies.

### 5.10 SurgiBot

The SPIDER Surgical System developed and improved by the TransEnterix Company,^33^, ^34^, ^35^ was later sold to Great Belief International Limited in China. SurgiBot is a bedside base robot that offers adjustable triangulation and multi‐quadrant movability with 3D‐enhanced HD vision. A preclinical trial involving urological procedures was reported in 2015.

### 5.11 PROCEPT

Aquablation (Aquatic ablation therapy) is one of the first endoscopic robots to perform surgery on its own, with the help of live ultrasound guidance. It was developed by the PROCEPT Company^36^ and rendered treatment of benign prostatic hypertrophy (BPH) a day‐case procedure, possible even in outpatient setups, with proven efficiency in short‐ and long‐term follow‐up. Its mechanism of action depends on the aqua beam system software with a surgeon's predefined contour, assisted by ultrasound‐calculated length, depth, and width of the planned resection.^37^ The WATER study showed that Aquablation therapy results were as effective as the gold standard transurethral resection of the prostate (TURP).^38^

### 5.12 EMARO

**FIGURE 5:**
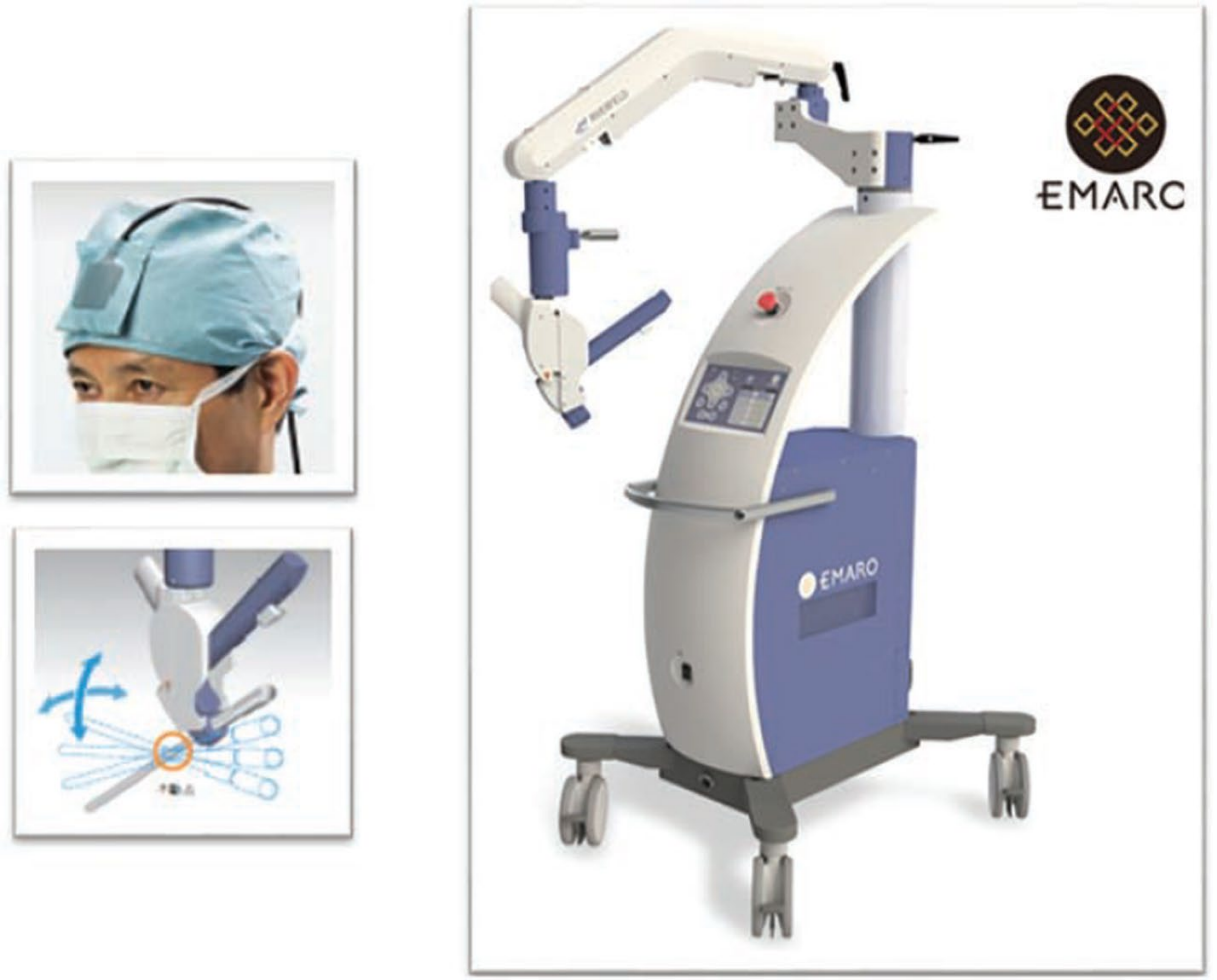
EMARO robot^3^, ^7^

EMARO is the first pneumatically powered endoscopic manipulator robot with an air‐pressure system (Figure 5).^13^, ^39^ It was developed by a Japanese company based in Tokyo. The operator drives the robot with the help of a head sensor.^39^, ^40^ We await more information on its clinical application and studies.

### 5.13 Avicenna Roboflex

Avicenna Roboflex (Elmed, Ankara, Turkey) was introduced for intrarenal surgery using a robotically driven flexible ureterorenoscope.^41^ It is an open console system with enhanced HD vision. The Avicenna Roboflex offers full control of flexible ureteroscopy, irrigation, LASER fiber, and fluoroscopy. The initially published clinical data are promising.^42^

## 6 DISCUSSION

Robotic‐assisted surgery established a new era and novel milestones in the history of surgery and its adoption among surgeons was extremely fast. It has reshaped the oncological and reconstructive surgeries throughout all surgical specialties. In part, these recent surgical advancements are the result of what the robot offers the surgeon: a tool set unlike any tools that proceeded it.^25^ Urologists exhibited precedent adoption of robotic surgery, especially for prostatectomy and partial nephrectomy.^43^ Even endourology had demonstrated growing use of robotic endourological surgeries.^42^ The intuitive surgery monopolized the surgical robot market over more than a decade, which gave the first systems a considerable competitive advantage. This advantage was the result of a strong market, trust of the surgical community, accumulative technical experience, and—last but not least—the numerous high‐quality clinical studies which have supported robotic systems and surgeries.^10^ As the patent rights expire, the robotic surgery market will be increasingly competitive, thus raising the bar for all robotic companies.

Although the da Vinci system propelled many robots to market, there has been no significant improvement in the console. The closed console system compromised the surgeon's awareness of his/her surroundings in the operative theater. In contrast, the ALFX system offers an open console system, which is a move in the right direction.^6^ Haptic feedback is a particularly important feature that is lacking in the current da Vinci system, while the Telelap ALF‐X took the initiative step of providing haptic feedback with their robots.^6^, ^16^ Given the emerging surgical trends of single‐site and natural orifice surgeries, we expect that robotic surgery will revolutionize these types of surgeries through the development of advanced surgical robots and minimally invasive technologies.^43^, ^44^ Many companies are currently developing their own surgical robots; however, many of these projects are confidential at this moment. This list of available surgical robots is non‐exhaustive, with each day bringing newly developed technology.

## 7 CHALLENGES TO NEW ROBOTIC SYSTEMS

New robotic companies face many challenges. The open surgical robot market is competitive, and cost is a significant factor in the success or failure of any one system. Companies whose robots are priced similar to existing trusted and tested robots may fail to sell their product. Unfortunately, the development of new, high‐quality technology is costly. Future studies on robot pricing are needed.

Another challenge is where to sell the robots. Currently, most high‐volume robotic surgery centers have reached their operative room capacity for accommodating robots. When new robotic companies target mid‐to‐small range hospitals, they do so at the risk of potentially disappointing initial clinical results, which may further reduce their market value.

Additionally, how will health insurance providers consider new surgical robots? Will these novel systems be covered by insurance? Will insurance companies classify all robotic surgery systems as “robotic,” regardless of the robot used, or will their classification schemes vary relative to the robot that is used? Many health insurance providers still do not cover robotic surgery by the da Vinci system, even given their long history in the field. Also, how will surgical robots affect medical litigation within the courts? Which robots be considered superior to other robots or to surgeons themselves? What if new robots demonstrate technical errors? These questions remain unanswered.

## 8 HINTS TO NEW COMPANIES

A strong marketing study and strategy are necessary for all new robotic companies, as their existing competitors have already established a strong market position, with enormous networks of distributors and technical support, earned trust among surgeons, and strong available clinical evidence. Surgeons are attracted to newer technologies, better vision, and easier learning curves. The best way to reveal the factors that motivate surgeons is to administer questionnaires to the members of robotic surgeon societies. Any new company should invest in the development of training modules and simulation software to ease the learning curve and increase robot adoption. Companies should avoid creating replicas of existing robots, and strive for creativity—from design to robotic technology execution. Too often, attempts to replicate existing products will produce inferior iterations. Developers should also realize the triangle of the three major beneficiaries of robotic surgery: surgeon, patient, and hospital. A senior surgeon values better technology that eases difficult surgeries. In contrast, junior surgeons value robots with standardized simulation and training modules, which help ease their learning curve and increase accessibility. Patients are also attracted to robotic surgery, as this technology is considered state‐of‐the‐art and is associated with reduced pain and scar size. Consequently, reaching patients via the media is of paramount importance. Meanwhile, hospital management sees value in reductions in the cost of the robot itself, reduced consumable instrument costs, and reduced hospital stay durations, ideally while increasing robotic surgery revenues.

## 9 CONCLUSION

This new era of robotic‐assisted surgery attracts both surgeons and patients. Robotic surgery has reshaped our surgeries over the last two decades, and robots are now used in almost in every surgical field. Still, as surgeons, we continue to look—with great interest—to new robotic companies that may be able to provide better robots in a more cost‐effective manner. Future studies that compare individual robotic systems are needed to further evaluate the strengths and weaknesses of each system, with an eye on the continued improvement of robotic surgery technology.

## CONFLICT OF INTEREST

None.

## AUTHOR CONTRIBUTIONS

Rha, Koon Ho: Protocol/project development/Data analysis/Manuscript writing/editing.

Almujalhem, Ahmad: Data collection or management/Data analysis/Manuscript writing/editing.
